# Microbial and immune faecal determinants in infants hospitalized with COVID-19 reflect bifidobacterial dysbiosis and immature intestinal immunity

**DOI:** 10.1007/s00431-023-05140-8

**Published:** 2023-08-09

**Authors:** Isabel Gutiérrez-Díaz, Miriam Sanz-Martinez, Ana Mª Castro, Marta Velasco Rodríguez-Belvís, Nathalie Carreira, Santiago Jiménez, Carmen Mangas, Macarena Queralt, Marta Herrador, Rafael Martín-Masot, Pablo Ferrer, Víctor M. Navas-López, Beatriz Espín, Rosaura Leis, Juan J. Díaz, Susana Delgado

**Affiliations:** 1 Department of Microbiology and Biochemistry of Dairy Products, Instituto de Productos Lácteos de Asturias (IPLA), Consejo Superior de Investigaciones Científicas (CSIC), Villaviciosa, Asturias Spain; 2 MicroHealth Group, Instituto de Investigación Sanitaria del Principado de Asturias (ISPA), Oviedo, Asturias Spain; 3 Gastroenterology and Nutrition Department, Hospital Universitario Infantil Niño Jesús, Madrid, Spain; 4 Paediatric Gastroenterology, Hepatology and Nutrition Unit, Complejo Hospitalario Universitario de Santiago de Compostela, Santiago de Compostela, Galicia, Spain; 5 Paediatric Nutrition Research Group, Institute of Sanitary Research of Santiago de Compostela (IDIS). CHUS-USC, Santiago de Compostela, Spain; 6 Paediatric Gastroenterology and Nutrition Section, Hospital Universitario Central de Asturias (HUCA), Oviedo, Asturias Spain; 7 Paediatrics, Primary Care Center “Otero,” Oviedo, Asturias, Spain; 8 Paediatric Gastroenterology Unit, Hospital Universitario Virgen del Rocío de Sevilla, Sevilla, Andalucia Spain; 9 Paediatric Gastroenterology and Nutrition Unit, Hospital Regional Universitario de Málaga, Málaga, Andalucia Spain; 10 Paediatric Service, Hospital Universitario y Politécnico La Fe de Valencia, Valencia, Comunidad Valenciana Spain

**Keywords:** Intestinal immaturity, Paediatric COVID-19, Infant microbiota, Bifidobacteria, Faecal cytokines, Faecal calprotectin

## Abstract

The coronavirus disease 2019 (COVID-19), caused by severe acute respiratory syndrome coronavirus 2 (SARS-CoV-2) has spread rapidly worldwide, seriously endangering human health. Although SARS‐CoV‐2 had a lower impact on paediatric population, children with COVID-19 have been reported as suffering from gastrointestinal (GI) symptoms at a higher rate than adults. The aim of this work was to evaluate faeces as a source of potential biomarkers of severity in the paediatric population, with an emphasis on intestinal microbiota and faecal immune mediators, trying to identify possible dysbiosis and immune intestinal dysfunction associated with the risk of hospitalization. This study involved 19 patients with COVID-19 under 24 months of age hospitalized during the pandemic at 6 different hospitals in Spain, and it included a comparable age-matched healthy control group (*n* = 18). Patients and controls were stratified according to their age in two groups: newborns or young infants (from 0 to 3 months old) and toddlers (infants from 6 to 24 months old). To characterize microbial intestinal communities, sequencing with Illumina technology of total 16S rDNA amplicons and internal transcribed spacer (ITS) amplicons of bifidobacteria were used. Faecal calprotectin (FC) and a range of human cytokines, chemokines, and growth factors were measured in faecal samples using ELISA and a multiplex system. Significant reduction in the abundance of sequences belonging to the phylum Actinobacteria was found in those infants with COVID-19, as well as in the *Bifidobacteriaceae* family. A different pattern of bifidobacteria was observed in patients, mainly represented by lower percentages of *Bifidobacterium breve*, as compared with controls. In the group of hospitalized young infants, FC was almost absent compared to age-matched healthy controls. A lower prevalence in faecal excretion of immune factors in these infected patients was also observed.

*Conclusion*: Hospitalized infants with COVID-19 were depleted in some gut bacteria, such as bifidobacteria, in particular *Bifidobacterium breve*, which is crucial for the proper establishment of a functional intestinal microbiota, and important for the development of a competent immune system. Our results point to a possible immature immune system at intestine level in young infants infected by SARS-CoV2 requiring hospitalization.

**Table:** 

**What is Known:** *• Although SARS‐CoV‐2 had a lower impact on paediatric population, children with COVID-19 have been reported as suffering from gastrointestinal symptoms at a higher rate than adults.* *• Changes in microbial composition have been described in COVID-19 adult patients, although studies in children are limited.*	
**What is New:** *• The first evidence that hospitalized infants with COVID-19 during the pandemic had a depletion in bifidobacteria, particularly in Bifidobacterium breve, beneficial gut bacteria in infancy that are crucial for the proper establishment of a competent immune system.* *• In young infants (under 3 months of age) hospitalized with SARS-CoV2 infection, the aberrant bifidobacterial profile appears to overlap with a poor intestinal immune development as seen by calprotectin and the trend of immunological factors excreted in faeces.*

**Supplementary Information:**

The online version contains supplementary material available at 10.1007/s00431-023-05140-8.

## Introduction

Coronavirus disease 2019 (COVID-19), caused by severe acute respiratory syndrome coronavirus 2 (SARS-CoV-2), has spread rapidly worldwide, seriously endangering human health. COVID-19 was declared a global pandemic in early 2020 by the World Health Organization (WHO), and to date, it has been diagnosed in more than 633 million people worldwide with 6,603,384 deaths (https://coronavirus.jhu.edu/data).

It has been observed that age is a differential factor in the prevalence and severity of SARS-CoV-2 [1]. The accumulated epidemiological and clinical data indicate that children, often more susceptible to respiratory virus infections [2], are less frequently and severely infected with SARS-CoV-2 than adults [1, 3, 4]. Most infected children were asymptomatic or had mild symptoms, being fever, cough, appetite loss, or gastrointestinal symptoms such as abdominal pain, nausea, diarrhoea, and vomiting the most frequent ones [5, 6]. In addition, some children with COVID-19 developed autoimmune and autoinflammatory complications, with features similar to Kawasaki disease, the so-called multisystem inflammatory syndrome in children (MIS-C) with significant digestive involvement [7, 8].

It has been estimated that the human body contains 10^14^ commensal microorganisms, tenfold the number of human cells, called microbiota, whose integrity is pivotal for human health. The majority of the microbiota reside in the gastrointestinal tract, taking part in the regulation of immune responses, inflammation status, and mucosal homeostasis, as well as in the defense against pathogens [9]. The establishment of gut microbiota takes place in the first stages of life, getting to relative stability by the age of approximately 2–3 years old, when its composition is more similar to the adult microbiota [10]. However, the microbial colonization can undergo fluctuations due to different factors such as type of delivery and breastfeeding, antibiotic administration, or hospital admission [11, 12]. In this context, while some studies have found changes in gut microbiota composition between patients infected with SARS-CoV-2 and healthy controls in adult populations [13, 14], the role of the gut microbiota in the susceptibility to COVID-19 infection in children remains unclear [8, 15, 16].

The aim of the present study was to examine gut microbiota composition and immune faecal factors in a paediatric population under 24 months of age hospitalized for COVID-19 disease, trying to identify potential relationships between intestinal dysbiosis and local inflammation associated with severe viral infection.

## Material and methods

### Participants

The study sample comprised 19 paediatric patients infected with SARS-CoV-2 under 24 months of age and 18 age-matched healthy controls. Patient recruitment was carried out in different public hospitals in Spain: “Hospital Universitario Central de Asturias” (Oviedo), “Complejo Hospitalario Universitario de Santiago de Compostela” (Santiago de Compostela), “Hospital Universitario y Politécnico La Fe” (Valencia), “Hospital Universitario Infantil Niño Jesús” (Madrid), “Hospital Universitario Virgen del Rocío” (Sevilla), and “Hospital Regional Universitario de Málaga” (Málaga). The recruitment period took place from October 2020 to July 2021. Inclusion criteria for patients’ selection were as follows: less than 24 months old, positive swab test for SARS-CoV-2 using reverse transcriptase quantitative polymerase chain reaction (qPCR) assay, and need for hospital admission. The control group was recruited by paediatricians in Spanish primary care centres at the time of scheduled well-child care visits, and inclusion criteria were as follows: (i) no previous antibiotic intake along the 3 months prior to sample collection, (ii) no clinical history of chronic and/or digestive tract related diseases, (iii) have not been diagnosed with COVID-19 by qPCR or antigen test, (iv) have not been in contact with people diagnosed or with suspicious of SARS-CoV-2 infection, and (v) do not present any symptoms of COVID-19 at the time of sampling.

Parents or legal guardians of all participants were informed of the objectives of the study, and they gave informed written consent. After that, stool samples were collected from each participant by its relative allowed to be at the hospital room. For that, families were provided with gloves, a sterile spoon and a stool sample tube. In the case of patients, the collection of samples was performed within the first 12 h after hospital admission. Clinical data was collected by clinicians and managed using Research Electronic Data Capture (REDCap) tools [17], hosted at “Sociedad Española de Gastroenterología, Hepatología y Nutrición Pediátrica” (SEGHNP) (redcap.seghnp.org) with assist from AEGREDCap Support Unit, shared with “Asociación Española de Gastroenterología” (AEG).

Ethical approval for this study was obtained from the Regional Ethics Committee for Clinical Research (Servicio de Salud del Principado de Asturias, no. 112/13) and from the Bioethics Committee of Consejo Superior de Investigaciones Científicas (CSIC) in compliance with the Declaration of Helsinki.

### Stool sample preparation

After stool collection, faecal samples were immediately frozen at the recruitment institutions. Transport to the Institute of Dairy Products of Asturias (IPLA-CSIC) was performed on dry ice in shipping biological containers type CTM03 according to UN3373 standard. At IPLA-CSIC, biological samples were stored at − 80 °C until use. Stool samples were thawed on ice for 30 min in a biosafety cabinet. Viral inactivation was performed with MagMAX Viral/Pathogen Binding Solution (Thermo Fisher Scientific Inc., MA, USA) for 10 min at room temperature in a relation 1:2 (w/v) [18]. Subsequently, stool samples were used or divided in aliquots for further analytical determinations.

### DNA extraction and quantification

Faecal dilutions (1:2) were homogenized in phosphate-buffered saline (PBS) to reach a dilution 1:10 for DNA extraction. This was based on the International Human Microbiome Standards (IHMS), protocol Q [19] using the QIAamp DNA Stool Mini Kit (Qiagen, Hilden, Germany) with slight modifications. A mechanical lysis was performed in a Fisherbrand Bead Will 24 homogenizer (Thermo Fisher Scientific Inc., MA, USA) with 3 cycles of lysis for 45 s each and leaving the samples on ice for 5 min between each treatment. The DNA was eluted and resuspended in 50 µl of molecular-biology grade water (Sigma-Aldrich, Saint Louis, USA), and stored at − 20 °C until use. DNA concentration was quantified in a Qubit fluorometer with dsDNA assay kits (Thermo Fisher, Waltham, USA).

### High-throughput sequencing of 16S rRNA gene amplicons and ITS region of rRNA genes amplicons of bifidobacteria

The 16S rDNA was amplified from the DNA of samples according to Milani et al. (2013) with primers pair “Probio_Uni/Probio_Rev” targeting the V3 region of the 16S rRNA gene, and 250 bp paired-end sequences were obtained using an Illumina MiSeq System (Illumina, San Diego, USA) at the spin-off of the University of Parma Genprobio srl (Italy) [20]. The sequences were processed using the Quantitative Insights Into Microbial Ecology (QIIME) software suite and were classified to the lowest possible taxonomic rank considered, using the SILVA database v. 132 as reference. In the same way, the internal transcribed spacer (ITS) region of rRNA genes of *Bifidobacterium* genus was amplified and subjected to high-throughput sequencing. For this, the primers “Probio_bif_uni” and “Probio_bif_rev” were used [21], together with an improved bifidobacterial ITS database and Genprobio personalized bioinformatic scripts. The raw sequences data were deposited in the Sequence Read Archive (SRA) of the NCBI (https://www.ncbi.nlm.nih.gov/sra) under BioProject ID: PRJNA911135 (sequence library IDs SAMN32161911 to SAMN32161927) and BioProject ID PRJNA914097 (sequence library IDs SAMN32320403 to SAMN32320420).

### Intestinal inflammatory biomarkers

#### Faecal calprotectin determination

Calprotectin levels were quantified using the commercially available enzyme-linked immunosorbent assay (ELISA) kit CALPROLAB™ (Calpro, Lysaker, Norway) according to the manufacturer’s instructions.

#### Faecal immune factors

The concentration of 27 cytokines, chemokines, and growth factors was determined using a Bio-Plex 200 system (Bio-Rad, Hercules, USA) and the Bio-Plex Pro Human Cytokine 27-plex Assay kit (Bio-Rad). Before analysis, faeces diluted tenfold (w/v) in PBS were centrifuged for 15 min at 20,000 g at 4 °C, and the supernatants were collected, diluted, and treated following the manufacturer’s protocol. Standards and samples were determined in duplicate. Data acquisition was performed with the Bio-Plex Manager 6.0 software and the standard curves fitted to a 5-parameter logistic regression.

### Statistical analyses

Statistical analyses were performed using IBM SPSS Statistics v.28.0.1 (IBM, Armonk, NY, USA). To examine the changes between patients and controls, we used the non-parametric *U*-Mann Whitney test and two-tailed probability values of *p* ≤ 0.05 were considered significant. In turn, medians, means, and interquartile ranges (Q1 and Q3) were represented in box and whisker graphics using the Origin Pro-2021 software (OriginLab, Northampton, MA, USA). Principal component analysis (PCA) was performed using R v.4.2.1. (“FactoMineR” and “Factorextra” packages).

## Results

### Participant characteristics

For the analysis of the results, the data was processed according to two groups of age: newborns or young infants including those between the age of 0–3 months, and toddlers, between 6 and 24 months of age. Information of age, gender, prematurity, mode of delivery, and type of feeding for both controls and patients is shown in Suppl. Table 1.

A total of 20 newborns were included, 11 patients with COVID-19, and 9 healthy controls. While patients in the newborns’ group had an average age of 1.9 ± 0.6 months, the average age of controls was 1.7 ± 0.8 months. On the other hand, the toddlers’ group was composed of 8 patients with COVID-19 and 9 healthy controls. The median age of patients in this group was 17 ± 7.7 months and 11 ± 4.8 months for controls. The hospital of origin, as well as comorbidities/previous chronic pathology, the main reason for admission, the days in hospital, and the treatment for each patient are shown in Table 1. No significant differences were found between patients and controls with respect to the variables type of delivery, type of feeding, and prematurity in the different age groups.

**Table 1:** Clinical history of the patients

Subject	Comorbidities	Symptoms at admission	Hospital days	Therapy in hospital	Hospital of origin
**Newborns (0–3 months)**					
Cov-P12	-	Fever	1	Paracetamol	LAFE
Cov-P15	-	Apnoea and rhinorrhoea	1	Paracetamol	HMIRUM
Cov-P17	-	Fever	1	-	HUCA
Cov-P19	-	Fever	1	Oxygen mask or nasal prongs	HIUNJ
Cov-P24	-	Fever	1	Antibiotic: gentamicin and ampicillin	HUVR
Cov-P28	-	Fever	1	Paracetamol	CHUS
Cov-P29	-	Fever	10	Paracetamol	CHUS
Cov-P33	-	Fever	2	-	HUCA
Cov-P35	-	Urinary tract infection	6	Antibiotic: gentamicin and ampicillin	CHUS
Cov-P36	-	Fever	2	Paracetamol	CHUS
Cov-P37	-	Fever	3	Paracetamol	CHUS
**Toddlers (6–24 months)**					
Cov-P5	-	Fever	2	Paracetamol	LAFE
Cov-P10	-	Urinary infection and cough	1	Antibiotic: amoxycillin/clavulanic acid	LAFE
Cov-P22	Focal epilepsy	Epileptic episode	1	Depakine, levetiracetam, clobazam and movicol	HIUNJ
Cov-P27	-	Fever and hypertransaminasemia	3	Intravenous fluid therapy and ibuprofen	CHUS
Cov-P30	PFAPA^a^ syndrome	Fever	1	Paracetamol and prednisone	CHUS
Cov-P31	-	Fever	1	-	HUCA
Cov-P32	-	Cough and shortness of breath	1	Antibiotic (azithromycin), salbutamol and oxygen mask or nasal prongs	HUCA
Cov-P41^b^	-	Respiratory infection with fever	3	Diazepam, midazolam, prednisone and oxygen mask or nasal prongs	HIUNJ

*HUCA* Hospital Universitario Central de Asturias (Oviedo), *CHUS* Complejo Hospitalario Universitario de Santiago (Santiago de Compostela), *LAFE* Hospital Universitario y Politécnico La Fe (Valencia), *HIUNJ* Hospital Universitario Infantil Niño Jesús (Madrid), *HUVR* Hospital Universitario Virgen del Rocío (Sevilla), *HMIRUM* Hospital Regional Universitario de Málaga (Málaga)

^a^PFAPA (periodic fever, aphthous stomatitis, pharyngitis, adenitis)

^b^Diagnosed as MIS-C

**Fig. 1:**
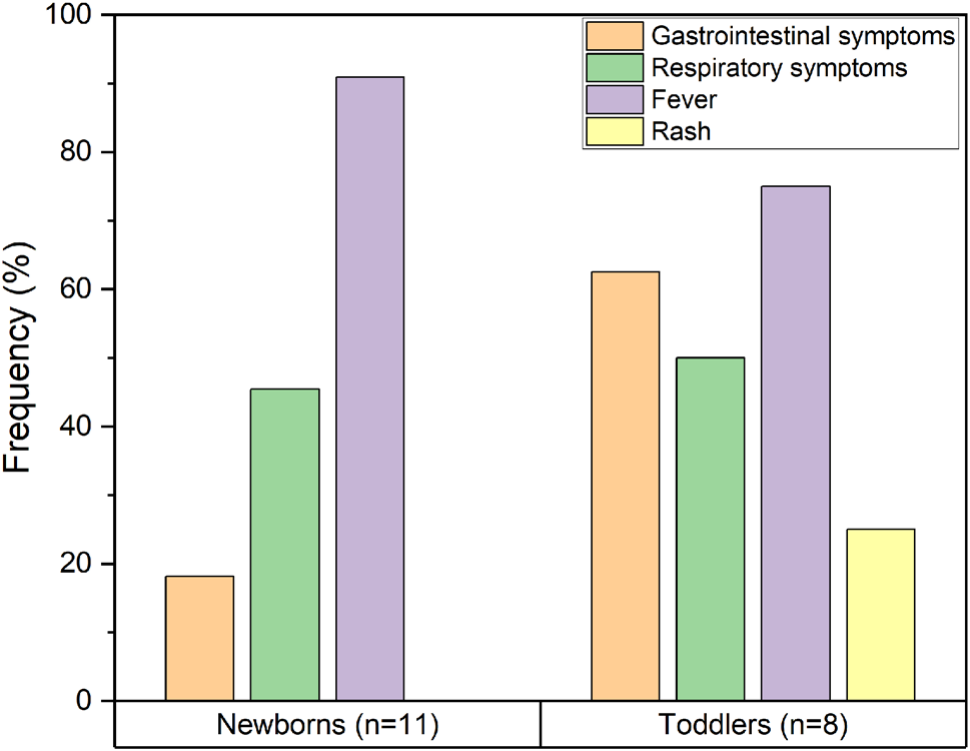
Main clinical characteristics found in COVID-19 patients according to age. The graphic shows the frequencies of each of the clinical symptoms found in patients. Newborns (0–3 months, *n* = 11). Toddlers (6–24 months, *n* = 8)

Fever was the main reason for admission in patients with COVID-19 in both groups. However, other clinical problems were observed (Fig. 1). Among newborns, 18.18% of patients presented digestive symptoms, with diarrhoea being the main clinical finding. On the other hand, 62.50% of toddlers’ cases showed digestive symptoms: abdominal pain (12.50%), nausea (25.00%), and diarrhoea (25.00%). Regarding other symptoms, respiratory problems were found in about 50.00% of patients (45.50% in newborns *versus* 50.00% in toddlers). It was observed that 25.00% of the patients in the toddler group presented skin alterations. (Fig. 1). In the newborns’ group 18.18% were treated with antibiotics; meanwhile, in the toddlers’ group, this percentage was 25.00% (Table 1). In this last group, 37.50% of patients received immunosuppressive therapy at hospital.

### Faecal microbiota and compositional profile

**Fig. 2:**
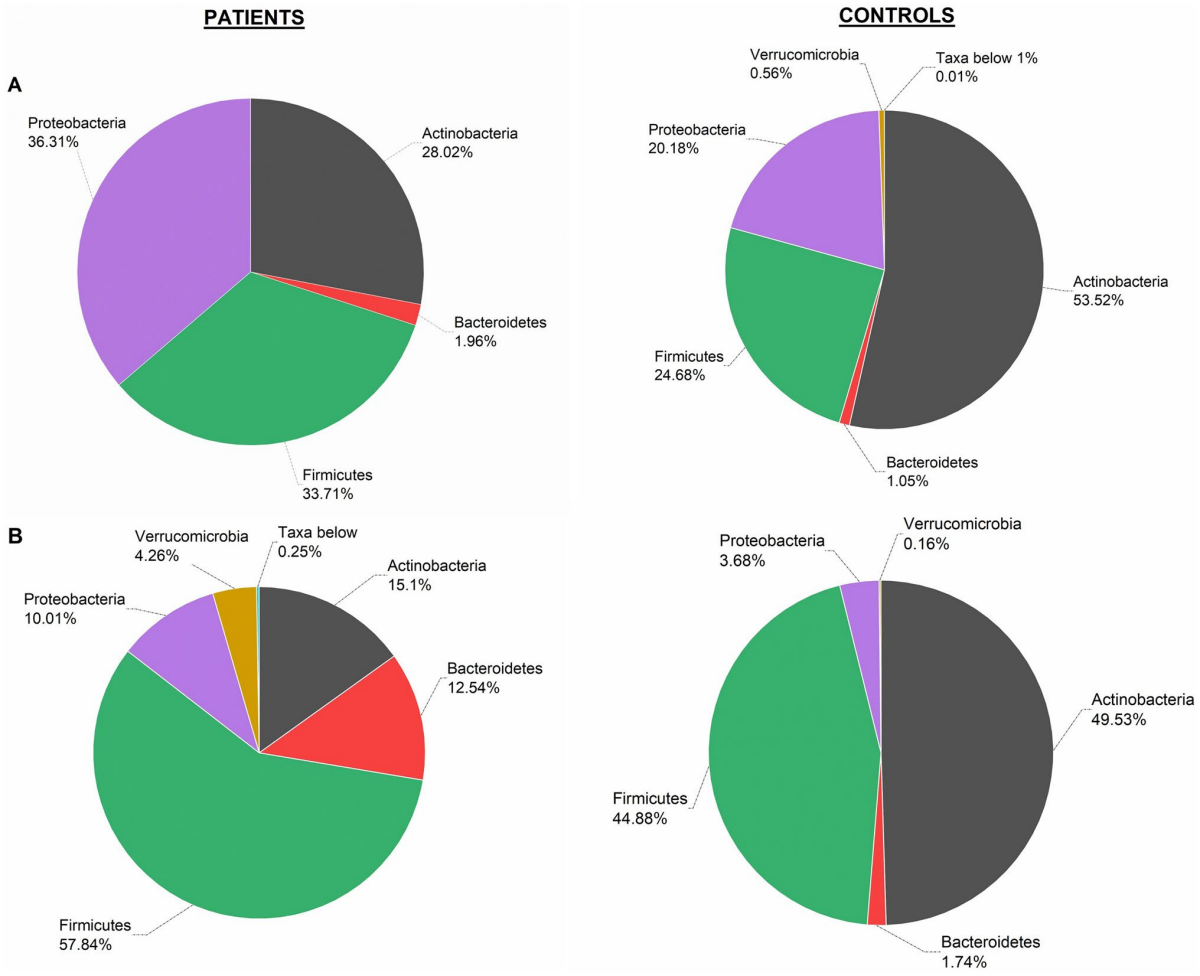
Differences in microbial relative abundance in faecal samples at phylum level between COVID-19 patients and the control group. **A** Newborns (0–3 months, 9 patients and 9 controls). **B** Toddlers (6–24 months, 8 patients and 9 controls). **p-value ≤ 0.01

Analysis of faecal microbial 16S sequences showed that Actinobacteria’ abundancy was lower in COVID-19 patients in both age groups (newborns and toddlers) as compared with controls (Fig. 2). In particular, in the newborn group, Actinobacteria phylum appeared reduced in COVID-19 infants (28.02 ± 32.76 patients *versus* 53.52 ± 28.69 controls), but no statistically significant differences were observed (*p*-value = 0.113). A significant reduction was observed in toddlers (*p*-value = 0.006), with lower Actinobacteria proportion in COVID-19 patients (15.10 ± 20.19) compared to healthy controls (49.53 ± 24.22) (Fig. 2B). On the other hand, while in the newborn patients the main microbial phyla were represented by Proteobacteria and Firmicutes followed by a low percentage of Bacteroidetes, in newborn controls, the microbial profile was distributed by Proteobacteria, Firmicutes, Bacteroidetes, and a reduced number of sequences belonging to Verrucomicrobia phyla (0.56% ± 1.19) (Fig. 2A). In contrast, looking at faecal microbial composition among toddlers (patients), the reduction of Actinobacteria provided a redistribution of the abundance of the rest of the phyla in comparison with controls, dominated by sequences of Firmicutes and Actinobacteria (Fig. 2B). Percentages of sequences of Proteobacteria, and Bacteroidetes, were higher in patients with COVID-19, although no significant differences were found.

In the newborns’ group, we observed that the most abundant families were represented by *Bifidobacteriaceae* and *Enterobacteriaceae* (Suppl. Table 2). Sequences belonging to the family *Bifidobacteriacea*e were more abundant in faecal samples from the control group (mean relative abundance 51.21%) compared with samples from patients with COVID-19 (mean relative abundance 25.84%). On the other hand, the relative abundance of *Enterobacteriaceae* family was higher in patients with COVID-19 than in controls (36.11% and 16.60%, respectively). In the toddlers’ group, a significant reduction in the proportions of sequences belonging to *Bifidobacteriaceae* family in patients (mean relative abundance 11.20%) compared to controls (mean relative abundance 44.34%) was observed (*p*-value = 0.004) (Suppl. Table 2). Relative abundance of *Enterococcaceae* family sequences was significantly higher in controls compared with patients (*p*-value = 0.027), without reaching percentages higher than 2% in any case.

At genus level, *Bifidobacterium* was the predominant genus in newborns, with a higher relative abundance in controls (51.21 ± 27.90) compared to patients (25.82 ± 32.51) (Suppl. Table 3). Moreover, a high percentage of assigned reads to the enterobacteria *Escherichia-Shigella* was observed in the samples of newborn patients (mean relative abundance 26.83%). In the toddlers’ group, we found a greater diversity of anaerobic genera belonging to different families, such as *Ruminococcaceae*, *Lachnospiraceae*, and *Bacteroidaceae* among others. The genus *Bifidobacterium* also showed a significant higher representation in the control group (*p*-value = 0.004).

**Fig. 3:**
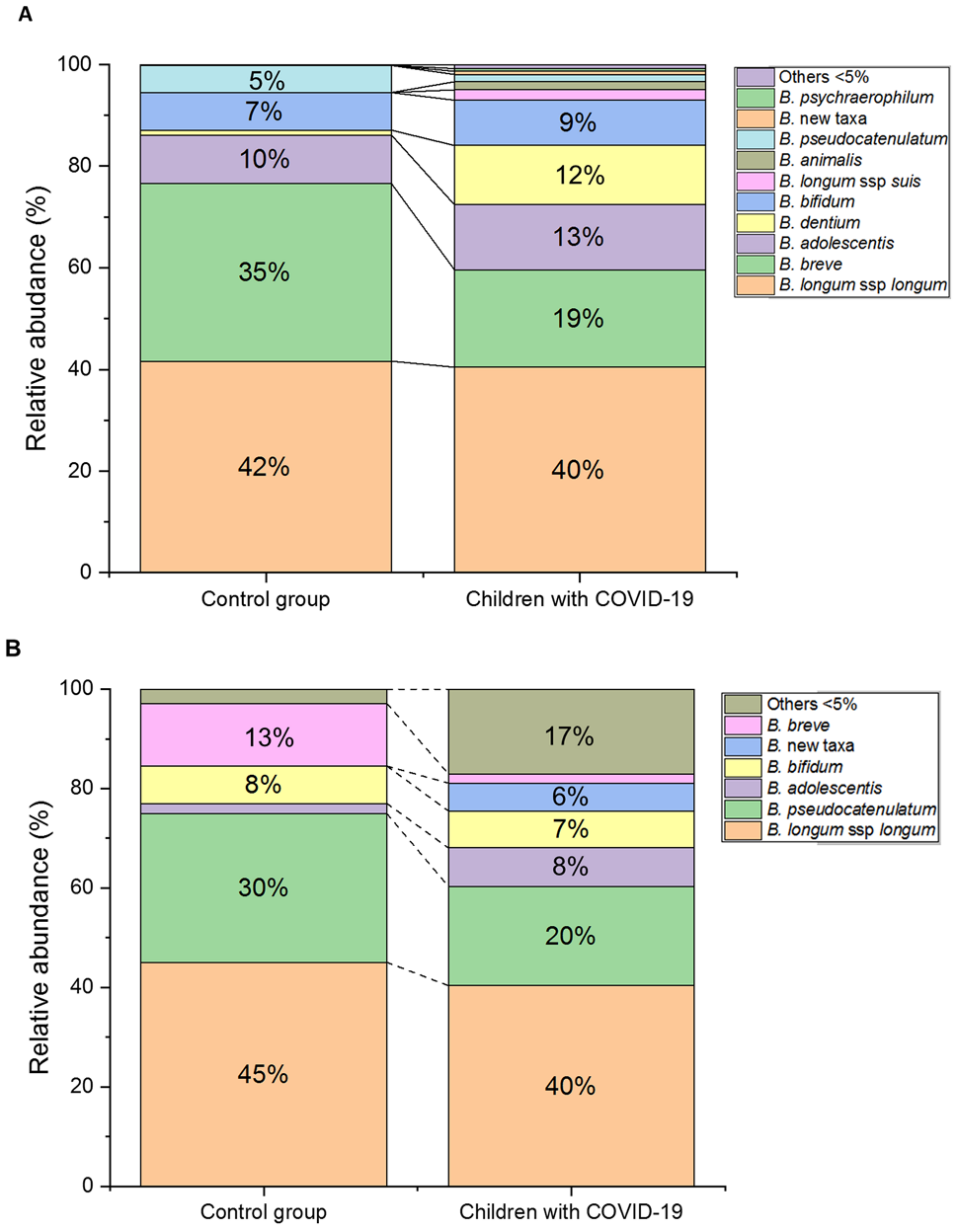
Profile of internal transcribed spacer (ITS) sequences of bifidobacteria represented as relative abundance in patients and controls in newborns (9 patients and 9 controls) (**A**) and toddlers (8 patients and 9 controls) (**B**). Data showed percentages of species of *Bifidobacterium* above 5% at least in one group

Therefore, we investigated whether the reduction of Actinobacteria observed for COVID-19 patients was associated with changes in the bifidobacterial profile. We analysed the presence and diversity of bifidobacteria species through sequencing the ITS region. As depicted in Fig. 3, a higher diversity of bifidobacteria in patients with COVID-19 was detected in both groups of age as compared to healthy children. In newborns, we observed that the majority of sequences corresponded to *Bifidobacterium longum* and *Bifidobacterium breve* species (Fig. 3A). In newborn patients, a slight reduction in *B. breve* and an increased number of *B. dentium* sequences was observed as the main characteristics. Also of note was the presence of an ITS profile characterized by other less abundant species that were not found in the healthy group, such as *B. longum* spp. *suis*, *B. animalis*, and other bifidobacteria not classified yet (Fig. 3A).

On the other hand, among toddlers, we found statistical differences at genus level. Specifically, patients showed reduced levels of *Bifidobacterium* through 16S sequencing (*p*-value = 0.004). Similarly, when we analysed the ITS profile, data showed that patients with COVID-19 had a greater diversity of bifidobacteria species than controls (Fig. 3B). Also, at this age range, reduction in the proportion of *B. breve* was also observed as compared with controls, together with an increase of sequences belonging to undetermined species of *Bifidobacterium* (new taxa).

A complementary analysis was carried out considering GI symptoms in the full sample studied: 19 patients and 18 healthy controls. Although no significant differences were found, results showed a differential microbial profile of the patients with GI characterized by Proteobacteria and Firmicutes as the major phyla, an increase in Bacteroidetes and a decrease in Actinobacteria sequences when compared to healthy controls and patients without GI symptoms (data not shown).

### Faecal immune biomarkers

#### Calprotectin levels

**Fig. 4:**
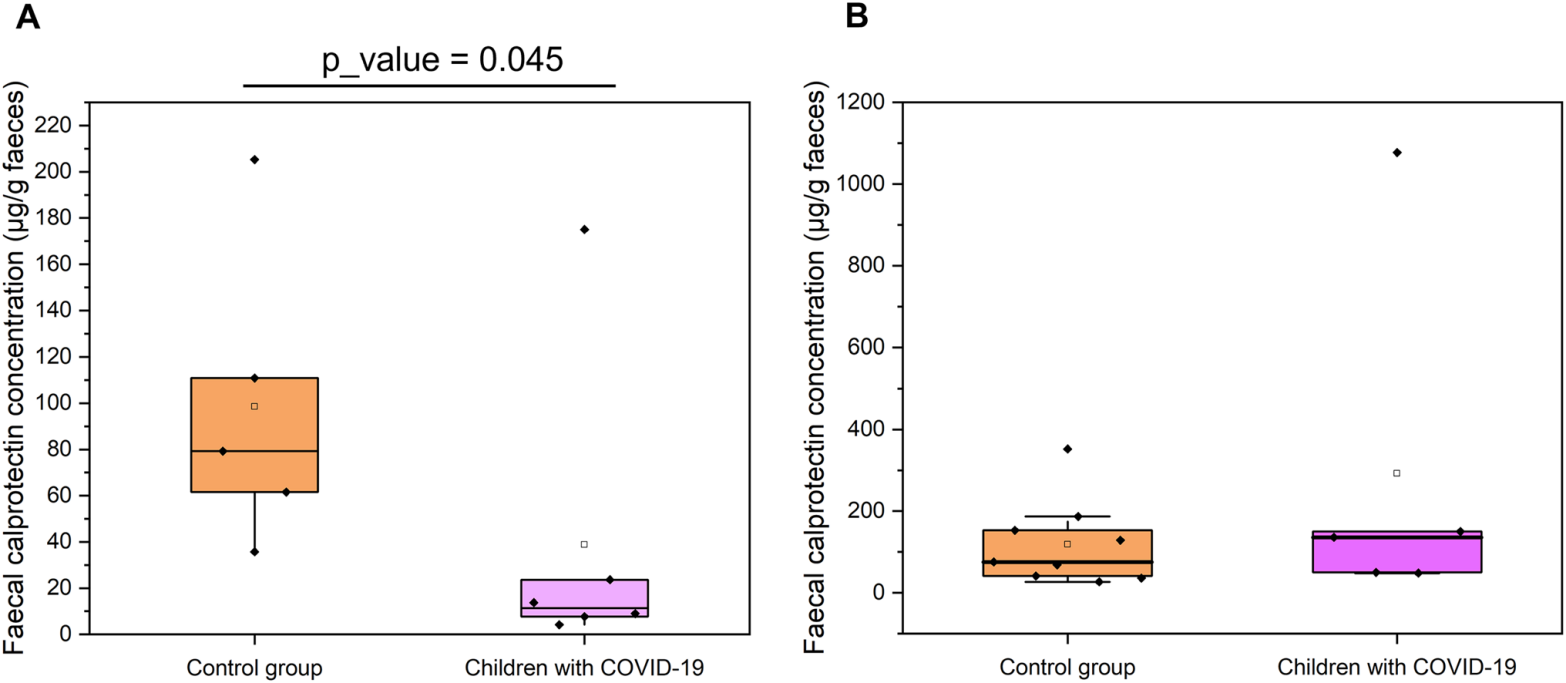
Calprotectin concentration (μg/g of faeces) in the faecal samples of the newborns (6 patients and 5 controls) (**A**) and toddlers (5 patients and 9 controls) (**B**). The lines inside the rectangle show the medians, and the whiskers show the maximum and minimum values. Comparisons were made with the *U*-Mann Whitney test to examine changes between patients and controls

As illustrated in Fig. 4A, faecal calprotectin (FC) concentration was significantly different among patients and controls in the group of newborns (*p*-value = 0.045). Contrary to what we expected the results showed that the mean FC was lower in patients (38.87 µg/g of faeces) than in healthy controls (98.53 µg/g). It should be noticed that the determination of FC was only possible in 4 patients and 5 controls, which considerably reduced the size of the newborns’ group.

On the other hand, the values of FC in toddlers did not show a statistical difference (*p*-value = 0.606). Patients had a FC concentration of 292.26 ± 441.28 µg/g (median ± standard deviation), whereas controls showed 118.84 ± 103.73 µg/g (Fig. 4B). Also here, we were unable to determine the calprotectin concentration in faeces for all participants of this age group due to the low quantity of faeces received (*n* = 5 for children with COVID-19) which could explain, in part, why we found no significant differences. Interestingly, the infant with COVID-19 who showed the highest FC level (1,077.12 µg/g of faeces) has been diagnosed with MIS-C following the WHO criteria [22]. In our analyses, this data was an extreme value (> 3 × interquartile ranges), so it was not considered for the statistical analysis.

#### Faecal immune factors

**Fig. 5:**
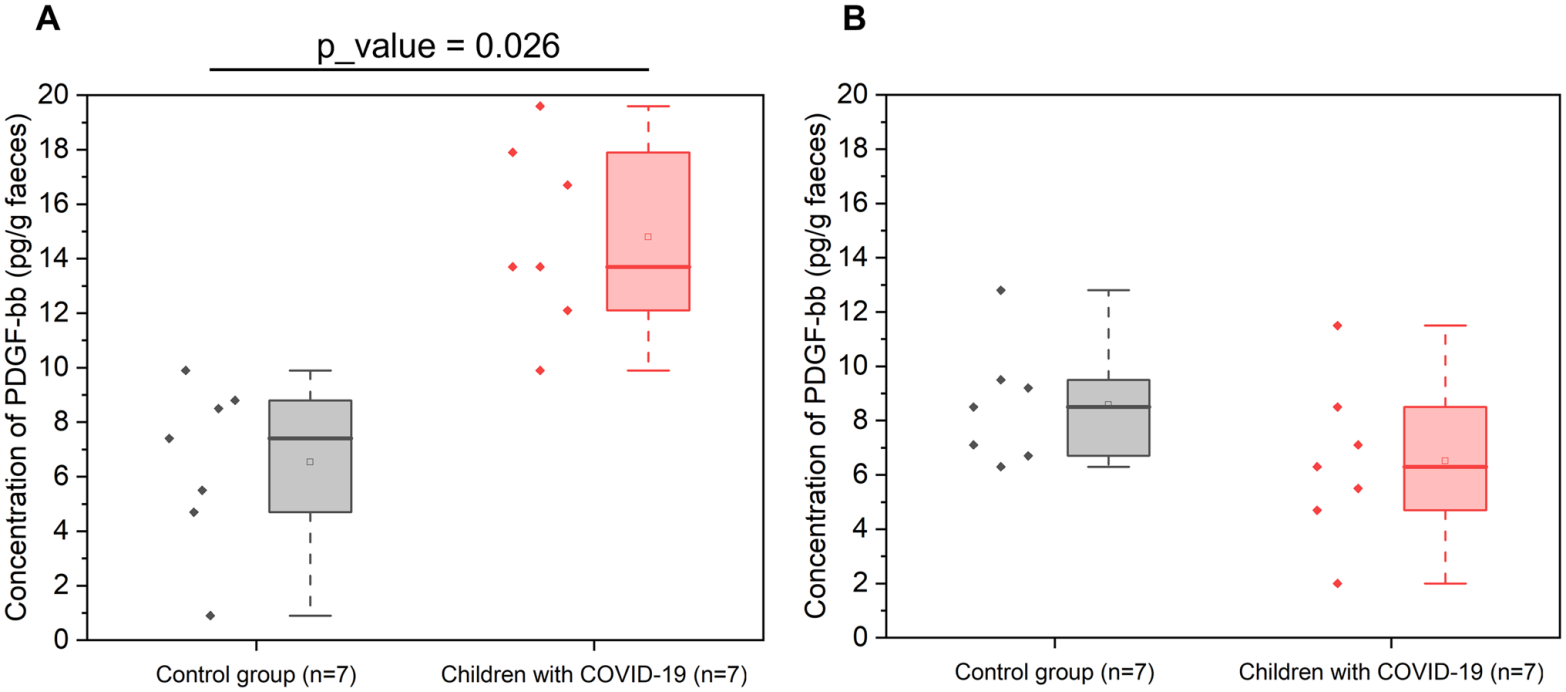
Plots showing faecal concentration of platelet-derived growth factor-BB (PDGF-bb) in COVID-19 patients and controls. **A** Newborns (0–3 months, 7 patients and 7 controls). **B** Toddlers (6–24 months, 7 patients and 7 controls). The lines inside the rectangle show the medians, and the whiskers show the maximum and minimum values. Comparisons were made with the *U*-Mann Whitney test to examine changes between patients and controls

A total of 27 cytokines, chemokines, and grown factors, measured as immune markets of intestinal barrier maturation and inflammation, were determined. For statistical analysis, cytokines that were detected in at least half of the individuals in one of the groups were evaluated. Out of 27 immune factors, 12 of them were detected, whereas the rest were undetectable or below the limit of quantification based on the standard curves of the immunoassays. Table 2 shows these faecal immune factors according to the group of age, with the exception of platelet-derived growth factor-BB (PDGF-BB) which is showed in Fig. 5.

**Table 2:** Concentration of immune compounds (pg/g faeces) in faecal samples

		**Newborns**		**Toddlers**	
Mediator	LOQ	Controls (*n* = 9)	Patients (*n* = 11)	Controls (*n* = 9)	Patients (*n* = 8)
IL-1b	0.001	0.100 (0.100–0.100) (*n* = 5)	0.100 (*n* = 1)	ND	ND
IL-1ra	0.146	2.450 (1.475–4.450) (*n* = 8)	2.600 (2.300–3.500) (*n* = 5)	2.300 (1.700–2.825) (*n* = 6)	0.750 (0.400–1.475) (*n* = 6)
IL-4	0.004	0.100 (0.100–0.125) (*n* = 4)	0.100 (0.100–0.150) (*n* = 3)	0.100 (0.100–0.100) (*n* = 2)	0.100 (*n* = 1)
IL-7	0.200	11.400 (6.000–11.400) (*n* = 5)	11.150 (7.525–14.775) (*n* = 2)	6.450 (5.175–7.725) (*n* = 2)	ND
IL-8	0.064	3.900 (2.600–4.500) (*n* = 5)	3.500 (2.400–10.150) (*n* = 3)	2.050 (1.250–3.075) (*n* = 4)	2.900 (2.700–14.000) (*n* = 3)
IL-9	0.087	0.900 (0.600–0.900) (*n* = 5)	1.000 (0.850–1.150) (*n* = 2)	0.400 (0.300–0.500) (*n* = 2)	0.500 (*n* = 1)
IL-10	0.018	0.500 (0.275–0.825) (*n* = 4)	0.250 (0.175–0.325) (*n* = 2)	ND	0.300 (*n* = 1)
IL-17	0.056	1.500 (0.400–2.550) (*n* = 7)	1.000 (0.300–1.000) (*n* = 5)	0.900 (0.675–1.375) (*n* = 8)	0.750 (0.500–1.075) (*n* = 6)
IP-10	0.144	2.500 (1.700–3.150) (*n* = 7)	2.150 (1.700–2.550) (*n* = 4)	1.550 (1.175–1.775) (*n* = 4)	9.350 (4.725–13.975) (*n* = 2)
MCP-1	0.038	0.300 (0.200–0.400) (*n* = 5)	ND	0.800 (0.350–1.225) (*n* = 4)	1.300 (*n* = 1)
MIP-1	0.005	0.750 (0.325–0.875) (*n* = 6)	0.550 (0.325–0.700) (*n* = 6)	0.350 (0.275–0.425) (*n* = 2)	3.000 (*n* = 1)

Data were expressed as medians and interquartile ranges (between brackets). The sample size of cytokines detected for each group is shown (n). In shadow is also shown those cases for which excretion was more frequent in controls. U- Mann Whitney tests were used to evaluate differences in concentrations among the age group assessed (controls *versus* patients)

*ND* non detectable, *LOQ* limit of quantification

For all quantified immune factors, but PDGF-BB, we did not find statistical differences. However, it should be noted that in both age groups, we observed a general trend towards a higher number of individuals excreting immune factors in controls compared to patients (Table 2), especially in the newborns. In this particular age group, a statistical difference in the levels of PDGF-BB was found (*p*-value = 0.026) between patients and controls, with newborns hospitalized for COVID-19 presenting higher faecal values (Fig. 5).

**Fig. 6:**
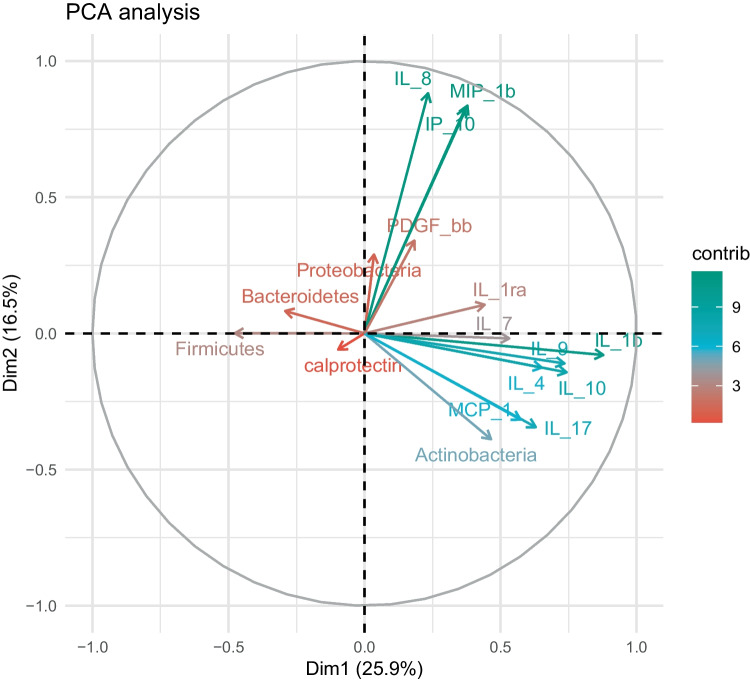
Principal component analysis (PCA) of faecal samples of the infants studied based on the microbiota data at phylum level and the faecal immune compounds determined

For multivariable analysis and comparison between patients and controls, PCA analysis with two components and all variables studied was performed. An association between Actinobacteria and most of the faecal immune factors was observed (Fig. 6) as they significantly correlated with the first same dimension, explaining 25.90% of the total variability.

## Discussion 

Several studies have been performed since the beginning of the pandemic, reflecting that SARS-CoV-2 infects not only the respiratory tract but also other epithelia such as the gastrointestinal mucosa where the angiotensin-converting enzyme 2 (ACE-2) receptors for the virus are found [23]. This direct viral infection may result in cytokine release and neutrophil activation at local intestinal level [24]. The intestinal epithelial cell damage caused by SARS-CoV-2 was thought to be associated with gastrointestinal symptoms, and particularly, children are reported to have more digestive symptomatology than adults [6]. Confirmed SARS-CoV-2 infection with gastrointestinal symptoms and changes in microbiota associated with COVID-19 severity have been reported in adults [25]. These types of studies in children are really scarce [8, 15, 16]. However, only in Spain, more than 1000 children were hospitalized due to COVID-19 [26]. In this work, we presented a multicentre study of infants with SARS-CoV-2 infection hospitalized in 6 Spanish hospitals. We observed that the presence of digestive symptoms was higher than 50%, at least in the case of infants from 6 to 24 months of age; however, we did not find statistical differences in the composition of the microbiota between patients with and without digestive symptoms.

Analysis of faecal microbial composition revealed a marked change with respect to control healthy infants in both groups (newborns and toddlers) that mainly consisted in a reduction in the percentage of sequences belonging to the Actinobacteria filum and *Bifidobacteriaceae* family. In agreement with our results, previous studies in children with COVID-19 reported a reduction in the abundance of Actinobacteria in the microbiota of patients compared to controls [8, 15]. Depletion of commensal bacteria (bifidobacteria) and increase of opportunistic pathogens have been also observed in adults [27]. In our work, among the genera *Bifidobacterium*, we also observed differences when comparing the ITS bifidobacterial profiles. A higher diversity of bifidobacterial, in terms of number of different species, was observed in patients with COVID-19 as compared with controls. In literature, it is reported that healthy children younger than 3 years of age possess low (alpha) diversity of the gut microbiota dominated by few bacterial species [28], representing *Bifidobacterium* the dominant bacterial genus [29]. In on work, relative abundance and number of species of bifidobacteria were different, with more presence of sequences assigned to *Bifidobacterium breve* in healthy control infants. *Bifidobacterium breve*, which is the main constituent of the intestinal microbiota of healthy newborns, is responsible for the development of intestinal biocenosis as well as for the activation of the immature immune system [30]. In fact, there are studies in animals’ models showing a direct activation of immature immunity by strains of this species [31]. As such, children with food allergy were observed to have decreased numbers of this species [32]. In brief, this bifidobacterial species that colonize the infant’s gut can induce epithelial barrier maturation and may protect against pathogenic bacteria [33]. In patients, we observed the presence of an ITS profile characterized by other bifidobacterial species that were not found in the healthy group, such as *B. animalis* or *B. dentium* which are known as cosmopolitan bifidobacterial species, not particularly associated to infancy [28].

FC is considered a useful diagnostic biomarker for intestinal mucosal inflammation, although their reference values are more clearly defined in adults than in children [34]. Calprotectin is an immunomodulatory, antimicrobial, and antiproliferative protein that is present in the cytoplasm of neutrophils, but also in the membranes of macrophages, in activated monocytes, and in mucosal epithelial cells, being of importance in defense mechanisms and physiological functions of the immune system [35]. However, no clear cut-offs have convincingly been established throughout infancy [36, 37]. Additionally, some studies have reported no clear consistence with digestive symptoms in COVID-19 [38, 39]. In this work, consistently with others previous observations [16], we did not find differences between values obtained in healthy and hospitalized toddlers ranging from 6 to 24 months of age. It is true that in this group of patients, a case of MIS-C with a FC over 1000 µg/g was included, which clearly reflects high inflammation and compromised gut barrier integrity which may make the surface of the enterocytes more susceptible to invasion by pathogens in the gut [40]. In fact, this patient presented an elevated colonization (45% relative abundance) by Gram-negative Proteobacteria as revealed by 16S sequence analysis (data not shown).

Unexpectedly, we found statistical differences between FC levels in newborns, ranging from 0 to 3 months of age, with higher values in healthy controls. This tendency was also described in babies under 1 year of age with cystic fibrosis in which controls had higher calprotectin values than patients [41]. This can point to an immature immune system at intestinal level with less neutrophils migration in hospitalized newborns with COVID-19. Velasco Rodríguez-Belvís and colleagues (2020) have already described that healthy babies between 1 and 5 months have elevated FC values probably due to the activation of the immune system after birth [34], with a high calling for neutrophils to the local mucosa and the activation of anti-defense mechanisms. Consistent with our observations, in adults, a dysfunctional immune response associated with gut microbiota dysbiosis has been proposed in severe COVID-19 [40, 42]. Trevelin and colleagues (2022) have already observed that the intestinal immune response is compromised in adults with severe COVID-19 [25]. These authors found a depletion of germinal centres in ileal Peyer´s patches and decreased potential B and T cell interaction and argue for a link with gut microbial dysbiosis.

Reinforcing this hypothesis, in this study, we found that faecal immune excretion (both anti and pro-inflammatory mediators) was more prevalent in healthy newborns than in those hospitalized with COVID-19. The only factor for which significant higher values were found in the faeces of patients was PDGF-BB. This factor, derived from platelets and related to vascular remodelling and angiogenesis, was shown in several studies to be strongly associated with severe COVID-19 [43, 44], although these results were observed in soluble plasma and in adults’ patients. This difference was not observed in the group of toddlers in whom the lower faecal cytokine excretion in patients was not so remarkable. It seems that in the small babies hospitalized with COVID-19, the intestinal immune system could not be completely mature and the mucosal lymphoid tissue might be still under development. Consistent with our theory, a higher frequency of faecal detection of immune factors in samples from healthy controls than in patients was shown in other work in infants (less than 18 months of age) with bronchiolitis [45]. In addition, local mucosal immune mediators in preterm infants were seen to be reduced or undetectable with similar faecal cytokine profiling technique [46]. However, in our cohort of study, there were not preterm infants (Suppl. Table 1).

On the one hand, SARS-CoV-2 may affect the intestinal microbiota promoting dysbiosis, as we have seen in this study in both neonates and toddlers. Nevertheless, the pre-existing microbiota may play an important role in determining individual susceptibility and resilience to COVID-19 as it has been suggested by other authors [40, 47]. Gut microbiota colonization in early infancy is linked with the immune system development and response [29]. Gut health at the time of SARS-CoV-2 infection may be critical and could be one of the reasons for the differences observed in the severity of symptoms among paediatric cases, from asymptomatic to requiring intensive care unit (ICU) admission. Underactive immune response, associated with microbial dysbiosis, such as that observed in infants in this work, may render the enteric mucosa more susceptible to invasion by pathogens, such as SARS-CoV-2, and therefore, it may affect disease progression, leading to potential complications.

This study was a preliminary work in infants younger than 2 years old, which explains some of its limitations, such as the reduced number of participants and the low quantity of sample recovered.This was derived to the difficulties experienced to access to this type of hospitalized patients during the pandemic due to the health alert situation and the critical studied population. All previous works with paediatric population with COVID-19 points how the sample size (with numbers varying from 9 to 13 patients) can be a limitation in finding relevant changes [15, 16]. Another potential bias in our work is the medical treatment received by the patients, and that despite samples were collected in the first deposition at hospital within the first 12 h after admission, we cannot dismiss some effect on the faecal parameters determined in the study. However, we consider that there is very few information regarding the affectation of the microbiota and the local immune reactivity in the GI tract in infants with SARS-CoV-2 infection, especially in those with moderate or severe manifestations. Unfortunately, in our work, it has not been possible to assess the different variants of the virus, which should be investigated in future studies to provide new hypotheses in the relationship between gut microbiota in infancy and COVID-19. More descriptive studies would contribute to fill this gap of knowledge and could give some light into the reasons leading to hospitalization.

## Conclusions

This study is presenting the first evidence of an aberrant microbiota profile together with a poor intestinal immune maturation trend in hospitalized infants under 3 months of age with COVID-19. Although with a reduced sample size our results point to a scenario that could facilitate severe disease caused by SARS-CoV-2. Additionally, *B. breve* has been seen as main depleted beneficial commensal in these patients’ gut. Novelty results connecting microbiota with SARS-CoV-2 in infant population will be useful to generate new hypotheses to test dietary strategies with probiotics in viral diseases.

### Supplementary Information

Below is the link to the electronic supplementary material.

Supplementary file1 (DOCX 46 KB)

## Data Availability

The datasets used and/or analyzed during the current study are available from the corresponding author on reasonable request.

## Abbreviations

ACE-2: Angiotensin-converting enzyme 2
AEG: Asociación Española de Gastroenterología
CSIC: Consejo Superior de Investigaciones Científicas
COVID-19: Coronavirus disease 2019
FC: Faecal calprotectin
GI: Gastrointestinal
ICU: Intensive care unit
ITS: Internal transcribed spacer
IHMS: International Human Microbiome Standards
IPLA-CSIC: Institute of Dairy Products of Asturias
MIS-C: Multisystem inflammatory syndrome in children
PBS: Phosphate-buffered saline
PDGF-BB: Platelet-derived growth factor-BB
PFAPA: Periodic fever, aphthous stomatitis, pharyngitis, adenitis
QIIME: Quantitative Insights Into Microbial Ecology
qPCR: Quantitative polymerase chain reaction
REDCap: Research Electronic Data Capture
SARS-Cov-2: Severe acute respiratory syndrome coronavirus 2
SEGHNP: Sociedad Española de Gastroenterología, Hepatología y Nutrición Pediátrica
SRA: Sequence Read Archive
WHO: World Health Organization
