# Supplementary material for: Microbial and immune faecal determinants in infants hospitalized with COVID-19 reflect bifidobacterial dysbiosis and immature intestinal immunity
Source: Eur J Pediatr. 2023 Aug 9;182(10):4633–45. doi: 10.1007/s00431-023-05140-8 (PMC10587250; doi:10.1007/s00431-023-05140-8)
Supplement: Supplementary file 1 — Supplementary file1 (DOCX 46 KB) [file 431_2023_5140_MOESM1_ESM.docx]

Supplementary Material

**Suppl. Table 1.** General characteristics of the participants in the study.

| Subject | Gender | Age (months) | Prematurity^1^ | Mode of delivery | Type of feeding^2^ |
| --- | --- | --- | --- | --- | --- |
| PATIENTS | | | | | |
| **Newborns (0- 3 months)** | | |  |  |  |
| Cov-P12 | Female | 2 | No | Vaginal | Breastfeeding |
| Cov-P15 | Female | 2 | No | Vaginal | Breastfeeding |
| Cov-P17 | Male | 1 | No | Vaginal | Mixed lactation |
| Cov-P19 | Female | 1 | No | Vaginal | Breastfeeding |
| Cov-P24 | Female | 2 | No | Vaginal | Mixed lactation |
| Cov-P28 | Female | 2 | No | Vaginal | Breastfeeding |
| Cov-P29 | Male | 3 | No | Caesarea | Breastfeeding |
| Cov-P33 | Female | 3 | No | Vaginal | Breastfeeding |
| Cov-P35 | Female | 1 | No | Vaginal | Bottle feeding |
| Cov-P36 | Female | <1 | No | Vaginal | Bottle feeding |
| Cov-P37 | Male | 1 | No | Vaginal | Breastfeeding |
| **Toddlers (6 -24 months)** | | |  |  |  |
| Cov-P5 | Female | 6 | No | Vaginal | Breastfeeding |
| Cov-P10 | Female | 24 | No | Vaginal | Breastfeeding |
| Cov-P22 | Female | 24 | No | Caesarea | Mixed lactation |
| Cov-P27 | Male | 12 | No | Vaginal | Mixed lactation |
| Cov-P30 | Female | 24 | No | Vaginal | Bottle feeding |
| Cov-P31 | Female | 10 | No | Vaginal | Bottle feeding |
| Cov-P32 | Male | 24 | No | Vaginal | Breastfeeding |
| Cov-P41^2^ | Male | 12 | No | Vaginal | Mixed lactation |
| CONTROLS | | | | | |
| **Newborns (0- 3 months)** | | |  |  |  |
| Cov-C10 | Male | 2 | No | Caesarea | Breastfeeding |
| Cov-C11 | Male | 1 | No | Vaginal | Bottle feeding |
| Cov-C12 | Male | 2 | No | Vaginal | Breastfeeding |
| Cov-C14 | Male | 1 | Yes | Caesarea | Breastfeeding |
| Cov-C15 | Male | 2 | No | Caesarea | Mixed lactation |
| Cov-C16 | Female | 3 | No | Vaginal | Breastfeeding |
| Cov-C17 | Male | 1 | No | Vaginal | Breastfeeding |
| Cov-C13 | Male | 1 | No | Vaginal | Breastfeeding |
| Cov-C18 | Male | 2 | No | Vaginal | Breastfeeding |
| **Toddlers (6 -24 months)** | | |  |  |  |
| Cov-C1 | Male | 16 | No | Vaginal | Mixed lactation |
| Cov-C2 | Male | 20 | No | Vaginal | Mixed lactation |
| Cov-C3 | Male | 12 | No | Vaginal | Mixed lactation |
| Cov-C4 | Male | 6 | No | Vaginal | Bottle feeding |
| Cov-C5 | Female | 6 | No | Vaginal | Breastfeeding |
| Cov-C6 | Female | 12 | No | Vaginal | Breastfeeding |
| Cov-C7 | Female | 12 | No | Vaginal | Breastfeeding |
| Cov-C8 | Female | 9 | No | Vaginal | Breastfeeding |
| Cov-C9 | Male | 6 | No | Vaginal | Breastfeeding |

^1^Prematurity was considered with less than 37 weeks of gestation. ^2^Breastfeeding was considered if it was along the first month of life, exclusively.

**Suppl. Table 2**. Differences in microbial relative abundance (%) at family level between patients with COVID-19 and the control groups. A) Newborns, B) Toddlers.

| A | **Newborns** | | | | | |  |
| --- | --- | --- | --- | --- | --- | --- | --- |
| Family | Patients (n=9) | | Controls (n=9) | | p-value | |  |
| *Bifidobacteriaceae* | 25.84± 32.51 | | 51.21 ± 27.90 | | 0.113 | |  |
| *Bacteroidaceae* | 1.21 ± 3.03 | | 0.25 ± 0.33 | | 0.546 | |  |
| *Staphylococcaceae* | 1.18 ± 3.19 | | 0.22 ± 0.27 | | 0.730 | |  |
| *Enterococcaceae* | 2.45 ± 6.08 | | 0.64 ± 0.99 | | 0.730 | |  |
| *Lactobacillaceae* | 0.21 ± 0.35 | | 0.76 ± 0.99 | | 0.190 | |  |
| *Streptococcaceae* | 9.32 ± 14.57 | | 8.78 ± 11.21 | | 0.730 | |  |
| *Clostridiaceae* | 8.79 ± 17.65 | | 1.38 ± 3.08 | | 0.136 | |  |
| *Lachnospiraceae* | 1.64 ± 3.04 | | 6.44 ± 16.78 | | 0.666 | |  |
| *Ruminococcaceae* | 0.45 ± 0.85 | | 2.82 ± 7.37 | | 0.931 | |  |
| *Veillonellaceae* | 8.99 ± 13.94 | | 2.26 ± 3.50 | | 0.340 | |  |
| *Enterobacteriaceae* | 36.11 ± 27.50 | | 16.60 ± 14.61 | | 0.063 | |  |
| B | **Toddlers** | | | | | |  |
| Family | | Patients (n=8) | | Controls(n=9) | | p-value | |
| *Bifidobacteriaceae* | | 11.20 ± 16.21 | | 44.34 ± 22.47 | | **0.004** | |
| *Prevotellaceae* | | 4.74 ± 8.05 | | 0.01 ± 0.02 | | 0.139 | |
| *Rikenellaceae* | | 1.04 ± 1.57 | | 0.04 ± 0.07 | | 0.423 | |
| *Tannerellaceae* | | 0.79 ± 1.62 | | 0.04 ± 0.05 | | 0.370 | |
| *Enterococcaceae* | | 0.01 ± 0.04 | | 0.73 ± 1.16 | | **0.027** | |
| *Streptococcaceae* | | 3.91 ± 4.30 | | 5.88 ± 9.84 | | 0.963 | |
| *Christensenellaceae* | | 0.54 ± 0.85 | | 0.10 ± 0.25 | | 0.481 | |
| *Lachnospiraceae* | | 31.40 ± 23.28 | | 25.41 ± 13.03 | | 0.888 | |
| *Ruminococcaceae* | | 17.05 ± 12.21 | | 7.54 ± 7.89 | | 0.139 | |
| *Erysipelotrichaceae* | | 0.89 ± 0.65 | | 2.38 ± 2.79 | | 0.139 | |
| *Acidaminococcaceae* | | 0.67 ± 1.71 | | 0.00 ± 0.00 | | 0.093 | |
| *Veillonellaceae* | | 0.69 ± 1.18 | | 1.96 ± 2.17 | | 0.093 | |
| *Enterobacteriaceae* | | 9.12 ± 16.08 | | 3.50 ± 5.37 | | 0.888 | |
| *Akkermansiaceae* | | 4.00 ± 10.99 | | 0.16 ± 0.32 | | 0.606 | |
| Data shown mean relative abundance ± standard deviation. U- Mann Whitney tests were used to evaluate differences in relative abundances among controls *versus* patients. Significant differences were considered with a p-value≤0.05. Only families with a mean relative abundance higher than 0.5% were presented. Only families that were detected in more than half of the samples in each group were considered for the analysis. | | | | | | | |

**Suppl. Table 3**. Differences in microbial relative abundance (%) at genus level between patients with COVID-19 and the control groups. A) Newborns, B) Toddlers.

| A | **Newborns** | | | |
| --- | --- | --- | --- | --- |
| Genus | Patients (n=9) | | Controls (n=9) | p-value |
| *Bifidobacterium* | 25.82 ± 32.51 | | 51.21 ± 27.90 | 0.113 |
| *Bacteroides* | 1.22 ± 3.03 | | 0.25 ± 0.33 | 0.546 |
| *Staphylococcus* | 1.18 ± 3.19 | | 0.22 ± 0.27 | 0.730 |
| *Enterococcus* | 2.45 ± 6.08 | | 0.64 ± 0.99 | 0.730 |
| *Lactobacillus* | 0.21 ± 0.35 | | 0.76 ± 0.99 | 0.190 |
| *Streptococcus* | 9.32 ± 14.57 | | 8.78 ± 11.20 | 0.730 |
| *Clostridium sensu stricto 1* | 6.81 ± 18.20 | | 1.12 ± 3.09 | 0.489 |
| *Clostridium sensu stricto 3* | 1.88 ± 2.36 | | 0.25 ± 0.48 | 0.113 |
| *Faecalibacterium* | 0.28 + 0.72 | | 0.88 ± 2.51 | 0.931 |
| *Veillonella* | 8.73 + 14.00 | | 2.24 ± 3.52 | 0.387 |
| *Enterobacter* | 4.98 ± 14.08 | | 1.30 ± 3.68 | 0.666 |
| *Escherichia - Shigella* | 26.83 ± 20.93 | | 9.00 ± 7.21 | 0.094 |
| B | | **Toddlers** | | |
| Genus | Patients (n=8) | | Controls (n=9) | p-value |
| *Bifidobacterium* | 11.20± 16.21 | | 44.34 ± 22.47 | **0.004** |
| *Collinsella* | 3.35 ± 4.17 | | 4.75 ± 6.45 | 0.888 |
| *Bacteroides* | 5.33 ± 7.47 | | 1.65 ± 2.34 | 0.423 |
| *Alistipes* | 1.03 ± 1.55 | | 0.04 ± 0.07 | 0.423 |
| *Parabacteroides* | 0.19 ± 1.62 | | 0.04 ± 0.05 | 0.370 |
| *Enterococcus* | 0.01 ± 0.04 | | 0.73 ± 1.16 | **0.027** |
| *Christensenellaceae R-7 group* | 0.53 ± 0.86 | | 0.09 ± 0.26 | 0.370 |
| *Eubacterium fissicatena group* | 0.87 ± 2.17 | | 0.61 ± 0.93 | 0.606 |
| *Eubacterium hallii group* | 0.53 ± 0.86 | | 0.94 ± 1.49 | 0.743 |
| *Ruminococcus gnavus group* | 2.92 ± 4.22 | | 4.75 ± 4.34 | 0.236 |
| *Anaerostipes* | 1.31 ± 1.71 | | 2.66 ± 3.42 | 0.541 |
| *Agathobacter* | 1.79 ± 3.36 | | 1.21 ± 2.28 | 0.481 |
| *Blautia* | 6.71 ± 6.88 | | 6.36 ± 4.01 | 0.888 |
| *Firmicutes* bacterium CAG 56 | 2.87 ± 3.21 | | 0.50 ± 0.72 | 0.074 |
| *Dorea* | 0.65 ± 0.63 | | 0.47 ± 0.78 | 0.481 |
| *Faecalibacterium* | 8.25 ± 8.35 | | 2.36 ± 3.56 | 0.093 |
| *Eisenbergiella* | 0.90 ± 0.93 | | 0.63 ± 0.90 | 0.541 |
| *Roseburia* | 0.97 ± 1.23 | | 0.48 ± 0.76 | 0.370 |
| *Ruminococcaceae* UCG 002 | 1.34 ± 1.92 | | 0.11 ± 0.32 | 0.167 |
| *Ruminococcaceae* UCG 014 | 0.30 ± 0.54 | | 0.09 ± 0.28 | 0.423 |
| *Ruminococcus* group 1 | 0.65 ± 1.26 | | 0.43 ± 1.15 | 0.236 |
| *Ruminococcus* group 2 | 1.23 ± 2.60 | | 0.47 ± 0.83 | 0.606 |

Data shown mean relative abundance ± standard deviation. U- Mann Whitney tests were used to evaluate differences in relative abundances among controls *versus* patients. Significant differences were considered with a p-value≤0.05. Only genera with a mean relative abundance higher than 0.5% were presented. Only families that were detected in more than half of the samples in each group were considered for the analysis.
